# Secondary Parotitis

**Published:** 1904-06-25

**Authors:** 


### Hospital Clinics.

### SECONDARY PAROTITIS.

Secondary parotitis is an incident which has been noted as a complication in a large number of pathological conditions. These conditions, on the surface at least, appear to be of very varied nature, and it is not easy to trace in them the common element on which the inflammatory development in the parotid gland depends. The most frequent cases would appear to be those which follow operations on the pelvic and abdominal viscera, and the suggestion naturally arises that here, at all events, the parotitis is of septic origin. There are, however, many facts which oppose this suggestion. In the first place, the parotitis by no means invariably terminates in suppuration; the swelling, heat, tenderness and other signs of inflammation subside and no further trouble ensues. Even when suppuration occurs, it is only in occasional instances that there are other indications of pyæmia; the parotitis remains the sole inflammatory event which disturbs the course of the convalescence. Often, too, there are no rigors and no decided rise of temperature. It is difficult in these circumstances to regard the parotitis as an expression of pyæmia in any of the ordinary or recognised forms of that condition. Again, it may be observed that in a number of instances the swelling of the parotid only makes its appearance some two to three weeks after operation, and when the condition of the abdominal wound and the general state of the patient are entirely satisfactory. In other instances the complication is an early event; it has been noted within two or three days of an abdominal operation. Fortunately, whether early or late, parotitis appearing after surgical interference with the abdominal or pelvic viscera is, for the most part, not attended with any very serious consequences. It may, of course, retard convalescence and cause a considerable amount of trouble, but the issue is usually quite satisfactory. An experience recently reported by Brennan Dyball,[1] however, proves that occasionally parotitis may determine a fatal result. The case was that of a male patient, aged 37 years, who had been operated on for a fairly acute but uncomplicated appendicitis. After the operation there was no anxiety about the abdominal condition and the patient appeared to be making satisfactory progress, the temperature having fallen to 99° F., when, on the fourth day, the right parotid gland became tender and swollen and the thermometer rose to 100·4° F. This was followed by the development of a series of abscesses, attended with sloughing, of the parotid, and in spite of the adoption of radical surgical measures and of the injection of anti-streptococcus serum and other forms of treatment, the patient gradually sank, and died 15 days after the operation and 11 after the appearance of the parotid bubo. There were no general or local signs of septic infection, and the suppurative condition was limited to the substance of the parotid and did not extend to the cellular tissue of the neck or other parts. Thus, whilst the unfortunate result distinguishes the case from other records, the general circumstances do not in any way differ from previous experiences. The parotitis developed and ran its course without evidence of septic developments either in the primary wound or in any other part of the body. The case, therefore, throws no light on the exact relationship of the parotitis to the initial event—in this instance an appendicitis treated by abdominal incision. Stephen Paget[2] has argued strongly against the pyæmic nature of the parotitis in cases, of the above order, and has collected a long list of cases from an analysis of which he predicates the existence of some special connection through the nervous system between the abdominal and pelvic viscera and the salivary glands. He suggests that lesions of these viscera may lead reflexly to a congestion of the parotid gland, which may in some instances proceed to inflammation or even to suppuration. Apart from the fact that it is after operations on the pelvic and abdominal viscera that parotitis most frequently occurs, there are other facts which may be quoted in support of Paget's thesis. Thus, parotitis has been known to follow the introduction of a pessary, to develop at each monthly period in a case of suppressed menstruation, to signalise the onset of pregnancy, and to result from an injury to the testicle. Various other abdominal conditions not attended by surgical operations have also been complicated by parotitis. G. S. Middleton[3] describes it in a case of fæcal tumour, Hilton Fagge[4] in a case of intestinal obstruction due to cancer of the sigmoid flexure, and H. D. Rolleston[5] in ulcer of the stomach treated by nutrient enemata. The last-mentioned, quoting from the records of St. George's Hospital, finds parotitis more frequent in the circumstances mentioned than in appendicitis or any other abdominal condition. Another case of interest in connection with Stephen Paget's view is one described by Mertens,[6] in which obstructive jaundice, due apparently to the entrance of a round worm into the bile duct, was complicated by a double suppurative parotitis. There is, therefore, much to be said for the suggestion that some special pathological relationship exists between the pelvic and abdominal viscera and the salivary glands. But there are other facts which indicate that this is too narrow a view from which to study the problem of secondary parotitis. Parotid inflammation sometimes, for example, occurs without the existence of any sub-diaphragmatic lesion. Murchison[7] noted it in typhus, and it has been several times described in enteric fever, and occasionally in influenza. Eldon Pratt[8] has recently observed a bilateral suppurative parotitis in a fatal case of acute generalised tuberculosis attended with purpura hæmorrhagica. Again, pneumonia has in some few instances been attended with parotitis, either simple, or suppurative and gangrenous. Such examples are sufficient to show that the suggestion of a special relationship between the abdominal contents and the parotid gland is not sufficient to cover the ground. It has been urged that in all the above conditions there is apt to be dryness of the mouth, and that the conditions favour the passage of organic or other irritants from the buccal cavity along Stenson's duct. If this were the true explanation, it is certainly surprising to find that parotitis is so rare a complication of febrile conditions. Pyrexia, and consequent dryness of the mouth, extending over prolonged periods, are amongst the most frequent of clinical experiences, yet it is only rarely that with them there is parotitis. Further, as pointed out by Stephen Paget, in the circumstances suggested the *socia parotidis* ought to be earlier and more frequently attacked than the body of the gland. But experience shows that this is not the case. Another suggestion[9] advanced in explanation of the occurrence of secondary parotitis is that the gland in various circumstances may take on an excretory function, and that the septic or other foreign materials which it then separates from the blood may "irritate" it into a condition of inflammation with or without suppuration. In all the conditions in which parotitis has been described there is at least the opportunity for the entrance of inflammatory or other abnormal products into the blood, and hence there is at least a possible case in favour of the theory last mentioned. It is also to be remembered that the parotid and other salivary glands may undoubtedly engage in the separation from the blood of foreign materials which have gained entrance to that fluid. This is seen in the elimination in the saliva of such remedies as mercury and iodine. And a further stage in the argument is reached when it is remembered that occasionally such elimination is attended by considerable swelling of one or both parotids. Some few instances are also recorded in which the same fact has been noted in cases of chronic lead poisoning. Altogether, the "excretory" theory would appear to include the whole of the facts, though it does not in any way help towards the recognition of the nature of the immediate cause of the inflammation, nor to the prevention of parotitis as a complication of abdominal and pelvic operations.

The question has sometimes been raised whether some of the cases described as examples of secondary parotitis are not in fact instances of mumps. Thus, Wm. Elder[10] had a patient with double parotitis after an operation for appendicitis, and found that a case of mumps had occurred in the house some seven weeks earlier, which gives, it must be admitted, a somewhat lengthened incubation period. On the other hand, the view has been advanced that a true secondary parotitis may prove infectious to persons coming in contact with the patient. Bertram Addenbrooke[11] records a double parotitis without suppuration in a boy of 13 years three days after abdominal section for suppurative peritonitis. An aunt of the patient visited the boy and kissed him during the progress of the parotitis. Three days later she developed double parotitis, and after a further interval of three days her sister fell a victim to the same condition. No cases of mumps could be traced in the neighbourhood. These will be recognised as very interesting experiences, and the possibilities they suggest should be borne in mind. But those who have had much experience of secondary parotitis will hardly believe that their cases have been instances of parotitis epidemica (mumps), and, whilst Addenbrooke's record is a suggestive one, it is hardly sufficient in itself to support the proposition that secondary parotitis carries a risk of infection by direct inoculation.

1 Brit. Med. Jour., April 30, 1904. 2 Ibid., 1887, vol. i., p. 613. 3 Clinical Records, 1894, p. 15. 4 Principles and Practice of Medicine (second edition), vol. ii., p. 304. 5 Lancet, August 8, 1903, p. 393. 6 Medical Review, vol. i., p. 116. 7 The Continued Fevers (third edition), p. 219. 8 Brit. Med. Jour., Sept. 28, 1901. 9 C. O. Hawthorne, Glasgow Med. Jour., vol. xliv. 10 Lancet, Jan. 19, 1901. 11 Ibid., Dec. 29, 1900.

